# The Mediating Effect of Insecure Adult Attachment on the Relationship between Smartphone Addiction and Self-Directed Learning in University Students

**DOI:** 10.3390/nursrep10020016

**Published:** 2020-12-01

**Authors:** Inhee Park, Sinhyang Kim, Yeonok Suh

**Affiliations:** 1 Department of Nursing, Shinsung University, Dangjin 31801, Chungcheongnam-do, Korea; kimsin210@shinsung.ac.kr; 2 School of Nursing, Soonchunhyang University, Cheonan 31151, Chungcheongnam-do, Korea; yeonok@sch.ac.kr

**Keywords:** adult attachment, self-directed learning, smartphone attachment, college students

## Abstract

(1) Purpose: This study aimed to explore the mediating effect of insecure adult attachment on the relationship between smartphone addiction and self-directed learning in university students. (2) Methods: In total, 235 university students participated in this study. Data analysis was carried out through a three-stage verification procedure: Sobel test using technical statistics, Pearson correlation, and multiple regression analysis. (3) Results: Smartphone addiction was positively correlated with insecure adult attachment, attachment anxiety, and self-directed learning, whereas self-directed learning had a significant negative correlation with insecure adult attachment and attachment anxiety. Insecure adult attachment had a mediating effect on the relationship between smartphone addiction and self-directed learning. (4) Conclusion: Higher levels of smartphone addiction indicated higher levels of insecure adult attachment and reduced self-directed learning ability. Therefore, while the prevention of smartphone addiction is critical for improving self-directed learning skills, programs should be developed to foster the formation of secure adult attachment among university students.

## 1. Introduction

### Background and Necessity of the Study

Self-directed learning has become a major means of survival for people to acquire the knowledge and skills necessary to face challenges in an increasingly complex and rapidly changing society on their own. The ability to cope with changes and to solve problems is regarded as a core competency, and self-direction is required for this [1]. Self-directed learning is a form of learning in which the learner selects and decides the entire process of education voluntarily, from whether to engage in learning to setting goals, selecting educational programs, and evaluating education [2]. However, smartphone addiction and insecure adult attachment [3] are becoming social issues as they are factors negatively affecting self-directed learning. Smart phones, a digital medium that has recently emerged with the rapid development of the Internet, are becoming a necessity for modern people living in the Information Age. A smartphone is a handheld computer that not only provides the conventional functions of a mobile phone but also enables its user to undertake tasks including communication, information searching, music, games, shopping, finance, video viewing, and even corporate work anytime and anywhere. In particular, by using mobile Internet that connects virtual spaces and various SNS (social network service), it is opening a new chapter in human relationship and communication beyond time and space [4]. Smartphones have become a learning medium more accessible to learners. Using a smartphone for learning is suitable for self-directed learning as it allows learners to self-adjust the pace to suit their learning abilities. However, students are often distracted from self-directed learning by various apps on their smartphones. Learning has negative impacts on important tasks, learning processes, and achievements [5]. Due to the use of various apps on smartphones, students spend less time on self-directed learning, which in turn negatively affects important tasks, learning processes, and achievements [3,5]. However, there is also the down side of this necessity—the prevalence of smartphone addiction is rising across all age groups [6]. According to the 2018 National Information Society Agency (NIA) survey on the status of smartphone overdependence, those at risk for overdependence in Korea stood at 19.1% in 2018, a percentage that had more than doubled from 2011, when it was at 8.4% [7]. Further, the prevalence of smartphone addiction is highest among university students, who have been shown to have the highest risk for overdependence on smartphones [7]. Moreover, previous research has shown that university students’ smartphone addiction and mental health have a negative relationship, such that students experience headaches, fatigue, insomnia, concentration disorders, and behavioral addictions (e.g., game addiction) [8]. Further, higher levels of smartphone addiction in high school students was correlated with lower levels of self-directed learning ability [5]. Smartphone addiction has been reported to negatively affect student’s learning satisfaction during self-directed learning, maladjustment in school life, academic stress, and poor academic performance [9].

Recently, social attention is focused on insecure adult attachment—a failure to form secure attachment with another human being—as a major factor influencing college students’ addiction [10].

The origin of attachment in adulthood is formed through relationships with parents, but attachment types can change due to events experienced after childhood, and as they grow up, they can move from parents to whom their attachment is as the primary caregiver to relationships such as friends and lovers [11]. Adult attachment is the result of the attachment process which is formed in adulthood by switching the attachment target from parents to friends or lovers [12]. Berman and Spering [13] also defined adult attachment as an attempt to maintain physical and emotional stability and intimacy through contact with certain people in adulthood. Bartholomew and Horowitz [14] argued that attachment formed in adulthood cannot be interpreted the same as attachment formed in infancy. Adults argued that a variety of attachment relationships, such as family, peers, and lovers, should be applied to distinguish them from the attachment type of the relationship they had with their parents in the infancy stage [14].

Adult attachment is classified into attachment anxiety and attachment avoidance factors. Individuals with attachment anxiety become emotionally anxious when they do not experience emotional stability, become sensitive to others’ reactions, and show fear of abandonment. Individuals with attachment avoidance feel uncomfortable in close relationships with other people and typically avoid other people out of fear [15]. Attachment disorders may affect addiction based on the overall quality of the relationship with people who provide one with physical and psychological stability [16,17]. Feelings of unease and insecurity could increase the likelihood of the development of obsessive behavior, which might lead to an increase in smartphone addiction [18]. However, those with insecure attachment can change their patterns of attachment by either experiencing an important event in the course of their development or by changing their object of attachment. Therefore, maintaining close relationships when forming various relationships in adulthood is important for the development of adult attachment [19].

University students have an opportunity to interact with and become attached to a variety of people, as they are in a stage of their social lives that favors the formation of broad interpersonal relationships. A previous study found that it is desirable to understand adolescents’ current patterns of relationships with intimate friends rather than to identify early patterns of attachment [20]. As for previous studies on smartphone addiction, insecure adult attachment, and self-directed learning ability, there are reports on the relationship between adult attachment and smartphone addiction [20]; on the relationship between university students’ adult attachment, self-directed learning, and career adaptability, confirming that adult attachment has a moderating effect between these factors [3]; and on the mediating effects of experiential avoidance on the relationship between insecure anxious attachment and smartphone addiction [21]. However, insufficient empirical evidence is available to explain the relationship between adult attachment, smartphone addiction, and self-directed learning. Based on the research results, the author intends to provide basic materials to prevent smartphone addiction and to help students lead a healthy and vibrant university life as an efficient way to improve university students’ self-directed learning skills.

## 2. Research Aims

This descriptive study aimed to identify the relationship between smartphone addiction, insecure adult attachment, and self-directed learning abilities in university students and to explore the mediating effect of insecure adult attachment on these variables.

## 3. Methods

### Research Participants

Participants were recruited from a Korean University using an advertisement on the school’s website. Only students who wished to participate in the research could access the online survey after providing consent to participate. The inclusion criteria were (1) first- to fourth-year university students who were over 19 years old, (2) smartphone users, (3) those who understood the purpose of the study and consented to participate, and (4) those who could communicate in and read Korean.

G*Power (v 3.1.9.4; Universität Kiel, Kiel, Germany) was used to calculate the number of participants [22]. To conduct multiple regression analyses, the program demonstrated that the minimum required number was 107 participants when the significance level was established at 0.05, statistical power was 0.95, effect size was 0.15, and two predictors were used (smartphone addiction and insecure adult attachment). The data collected from all participants were included (*n* = 235) in data analysis, and we gathered a sufficient sample size based on the G*Power results.

## 4. Research Tools

### 4.1. Smartphone Addiction

The study used a scale developed by the National Information Society Agency [23,24] to measure smartphone addiction. Its subsections included 15 questions: daily life disorder (five questions), directivity of the virtual world (two questions), withdrawal (four questions), and tolerance (four questions). Participants responded to the questions based on a 5-point Likert scale (1 = *not at all*, 5 = *very much*), with higher scores indicating stronger smartphone addiction. The composite score for the scale was based on the mean score of the 15 questions. During development of the smartphone addiction tool, it showed reliability, with a Cronbach’s α of 0.88. For this study, Cronbach’s α was 0.83.

### 4.2. Insecure Adult Attachment

To measure insecure adult attachment, the Korean version of Experience in Close Relations-Revised (ECR-K), developed by Brennan [25] and translated by Kim [26], was used. Its subsections had 36 questions: attachment anxiety (18 questions) and attachment avoidance (18 questions). Participants responded to the questions based on a 5-point Likert scale (1 = *not at all*, 5 = *very much*), with higher scores indicating higher levels of insecure adult attachment. The composite score for the scale was based on the mean score of the 36 questions. During development of the translated tool in Kim’s study [26], the measure demonstrated reliability, with a Cronbach’s α of 0.89. For this study, Cronbach’s α was 0.89 (attachment anxiety Cronbach’s α was 0.86, and attachment avoidance Cronbach’s α was 0.87).

### 4.3. Self-Directed Learning Skills

To measure self-directed learning skills, the tool from Guglielmino and Guglielmino [27], the Self-Directed Learning Readiness Scale (SDLRS), which was verified for appropriateness by Kim [28] through exploratory factor analysis, was used. Its subsections had 22 questions: openness (five questions), concept of the self (five questions), passion (four questions), time management (four questions), and responsibility acceptance (four questions). Participants responded to the questions based on a 5-point Likert scale (1 = *not at all*, 5 = *very much*), with higher scores indicating higher levels of self-directed learning ability. The composite score for the scale was based on the mean score of the 22 questions. The tool was shown to be reliable in Kim’s study [28], having a Cronbach’s α of 0.94. For this study, Cronbach’s α was 0.87.

### 4.4. Data Collection Method and Ethical Considerations

All subjects gave informed consent for inclusion before they participated in the study. The study was conducted in accordance with the Declaration of Helsinki, and the protocol was approved by the ethics committee of SUNMOON University (IRB No: SM-202009-062-2) After explaining the contents and objectives of the research through a public notice related to the study development and possibility of participation, those who wished to participate accessed the URL specified in the notice and confirmed if they consented to participate, and only those who consented were able to participate in the online survey.

## 5. Data Analysis

For analysis of the collected data, IBM SPSS Statistics 24.0 (IBM, Chicago, IL, USA) Program was used. Descriptive statistics were reported on the general characteristics and other participant variables, while Pearson’s correlations were analyses to examine the relationship between smartphone addiction, insecure adult attachment, and self-directed learning abilities. To assess the mediating effect of insecure adult attachment on the relationship between smartphone addiction and self-directed learning ability, the study used Baron and Kenny’s [29] three-stage mediation effect verification process using multiple regression. The Sobel test was conducted to check for statistical significance.

## 6. Results

### 6.1. General Characteristics of the Participants

The general characteristics of the participants are shown in Table 1. Among the 235 students surveyed, 160 were women (68.1%) and 75 were men (31.9%). Smartphone daily usage time was between 4 and 7 h for 139 participants (59.1%), less than 4 h for 83 participants (35.3%), and more than 8 h for 13 participants (5.5%). Regarding the importance that participants gave to their smartphones, 180 reported that they were “important” (76.6%), 55 replied “neutral” (23.4%), and no participants reported that their smartphones were “not important.” Regarding whether the participant was addicted to smartphones, 102 answered “yes” (43.4%) and 133 responded “no” (56.6%).

### 6.2. Participants’ Smartphone Addiction, Insecure Adult Attachment, and Self-Directed Learning Ability

The participants’ mean smartphone addiction score were 2.52 (*SD =* 0.63) out of 5 points, and their mean insecure adult attachment score was 2.78 (*SD =* 0.28) out of 5 points. The participants’ average scores for each subscale of the insecure adult attachment tool were 2.64 (*SD =* 0.47) for attachment anxiety and 2.94 (*SD =* 0.30) for attachment avoidance. Participants’ mean self-directed learning ability scores was 3.37 (*SD =* 0.31) out of 5 points (Table 2).

### 6.3. Correlations between Smartphone Addiction, Insecure Adult Attachment, and Self-Directed Learning Ability

Smartphone addiction had significant correlations with insecure adult attachment (*r* = 0.47, *p* < 0.001), attachment anxiety (*r* = 0.46, *p* < 0.001), and self-directed learning ability (*r* = −0.34, *p* < 0.001). Self-directed learning ability had a significant negative relationship with insecure adult attachment (*r* = −0.33, *p* < 0.001) and attachment anxiety (*r* = −0.39, *p* < 0.001; Table 2).

### 6.4. The Mediating Effect of Insecure Adult Attachment on Smartphone Addiction and Self-Directed Learning Ability

Before validating the mediating effect, the regression model was validated and the autocorrelation of the error was tested using Durbin–Watson, which was independently shown as 2.12. The variance inflation factor stayed between 1.00 to 1.19. As the results were below 10, there was no problem with the mediating effect and there would not be any problem with multicollinearity.

**Figure 1:**
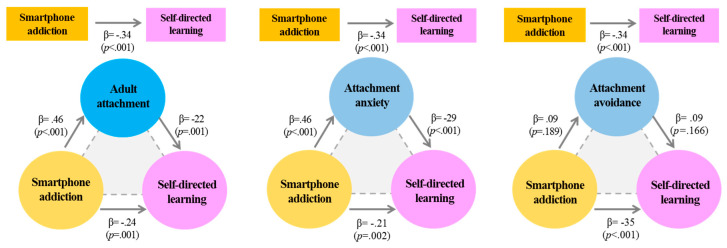
Scheme showing the participants’ adult attachment’s mediating effect on the relationship between smartphone addiction and self-directed learning. β, standardized regression coefficient.

The results of this study verified the mediating effect of insecure adult attachment in the process of smartphone addiction affecting self-directed learning ability, which is shown schematically in Figure 1 and Table 3. The first stage of analysis showed that the independent variable smartphone addiction had a significant effect on the mediating variable insecure adult attachment (β = 0.46, *p* < 0.001).

In stage 2, the independent variable smartphone addiction had a significant effect on the dependent variable self-directed learning ability (β = −0.34, *p* < 0.001).

In stage 3, smartphone addiction and insecure adult attachment were input as independent variables and self-directed learning ability was input as a dependent variable; the analysis showed that smartphone addiction (β = −0.24, *p* = 0.001) and insecure adult attachment (β = −0.22, *p* = 0.001) had significant effects on self-directed learning ability. However, the regression coefficient decreased from −0.34 in stage 2 to −0.24 in stage 3, indicating that insecure adult attachment had a partially mediating effect on the studied relationship. The results of the Sobel test were significant (*Z* = 2.05, *p* = 0.002).

In the subsections of insecure adult attachment, the mediating effect was statistically significant only for attachment anxiety. In stage 1 of the analysis, smartphone addiction was significantly associated with attachment anxiety (β = 0.46, *p* < 0.001) and smartphone addiction (β = −0.21, *p* = 0.002), whereas attachment anxiety (β = −0.29, *p* < 0.001) was significantly related to self-directed learning ability. In other words, with attachment anxiety as a mediating variable, the regression coefficient decreased from −0.34 in stage 2 to −0.21 in stage 3, showing that attachment anxiety had a partial mediating effect on the studied relationship. The results of the Sobel test were significant (Z = −3.80, *p* = 0.000). However, the mediating effect of attachment avoidance was not significant (Z = 0.96, *p* = 0.34).

## 7. Discussion

This study analyzed the mediating effect of insecure adult attachment (attachment anxiety and attachment avoidance) on the relationship between smartphone addiction and self-directed learning ability in university students. Overall, smartphone addition, self-directed learning, and adult attachment behaviors were significantly related to one another. Further, the analyses suggested that adult attachment partially mediated the relationship between smartphone addiction and goal-directed learning.

First, the analysis of the correlation between all major variables showed that higher levels of smartphone addiction were related to lower self-directed learning ability. These results corroborate previous studies that reported lower self-directed learning skills for university students who have higher levels of smartphone addiction [5,9]. These results also demonstrate the problems caused by smartphone addiction, consistent with previous research showing that university students are regularly exposed to many types of smartphone applications [30], which facilitates this addictive process. Some previous studies illustrated the benefits of smartphones in the promotion of self-directed learning, as they allow for learning applications to be accessed and utilized anywhere [31]. Nevertheless, other studies suggested that smartphone overuse can also harm self-directed learning [30,32]. Thus, most research suggests that smartphone addiction should be prevented in order to improve self-directed learning skills.

The present study also found that smartphone addiction was positively correlated with the levels of insecure adult attachment and attachment anxiety, according to existing research showing a significant positive correlation between smartphone addiction and the two variables from insecure adult attachment: attachment anxiety and attachment avoidance [20,33]. Given the present results and the existing body of research, insecure adult attachment and smartphone addiction should be investigated more thoroughly in future research.

Moreover, our findings showed that higher levels of insecure adult attachment indicated lower self-directed learning ability and that higher levels of attachment anxiety indicated lower self-directed learning ability, which is consistent with the findings of Kim that insecure adult attachment was positively correlated with self-directed learning ability [3]. Moon, Bak, and Yang similarly reported that students with secure attachment have better self-directed learning skills than those with insecure attachment [34]. As such, insecure adult attachment, smartphone addition, and self-directed learning are closely related. Therefore, various institutional improvement measures by colleges are urgently needed to ensure that college students’ smartphone addiction does not inhibit their self-directed learning abilities and to help them form secure adult attachment

Second, insecure adult attachment showed a significant mediating effect on the relationship between smartphone addiction and self-directed learning ability. The results of the current study are similar to the results of Kim’s study that reported a mediating effect of university students’ insecure adult attachment on the relationship between self-directed learning ability and career adaptability [3]. This confirms the hypothesis that higher levels of smartphone addiction may lead to more anxiety and fear related to insecure adult attachment, eventually leading to reduced self-directed learning ability. Through an indirect pathway, a previous study showed that a satisfactory attachment experience in university life can have a positive effect on the development of an individual’s character and on the formation of self-identity. However, a difficult attachment experience can lead to a lack of motivation to study, to dissatisfaction, to psychological conflict, and to depression [35]. In addition, a prior study on college students reported that insecure adult attachment had mediating effects [36]. Similarly, insecure adult attachment of college students has important implications in view of recent social issues. Smartphones are regarded as more than an everyday necessity, and as people form significant emotional attachments with this medium, the seriousness of smartphone addiction in students has been pointed out [3]. Therefore, to improve the self-directed learning abilities of college students, it is necessary to highlight the importance of secure adult attachment. Therefore, adult attachment seems to be a critical aspect of a university student’s life, suggesting the need to highlight and apply the concept of secure adult attachment to improve self-directed learning skills for university students who are at risk of smartphone addiction. However, as the population that was studied was limited to one university in South Korea, there may be limitations in generalizing and applying the research results; thus, future research is needed to replicate the present findings with other populations.

Additionally, the results showed a significant mediating effect of attachment anxiety when analyzing the relationship between smartphone addiction and self-directed learning ability. People with attachment anxiety are known to spend a great deal of time worrying about what other people think, as they have doubts about being loved and want to be excessively loved [37,38]. Further, those with attachment avoidance generally refuse or fear interpersonal relationships and avoid close relationships. In that regard, university students tend to use social networks, such as Kakao Talk and Facebook, as communication tools to engage in immediate interactions. In such relationships, the students’ feeling of belonging to the community, which is promoted through smartphone apps, can help them maintain and form personal relationships, but as the active interactions become increasingly habitual, smartphones may be used excessively. Thus, the desire to be recognized and loved by others and the anxiety over being abandoned by others may contribute to the exacerbation of smartphone addiction, which ultimately may affect one’s self-directed learning ability. Therefore, to help university students enhance their self-directed learning and diminish their smartphone addiction, awareness and consideration of secure adult attachment is necessary.

In summary, the results of this study and previous studies show that it is necessary to develop an attachment-building program and to create a correct learning environment to allow students to healthily utilize their smartphones in their daily lives. This should be performed to ensure that university students achieve a secure adult attachment in their relationships, thereby preparing them to advance in their self-directed learning abilities, which is a core competency for the current labor market.

## 8. Conclusions

This study showed that smartphone addiction among university students is a factor that is negatively associated with self-directed learning ability, and that insecure adult attachment is another critical psychological variable that is correlated to smartphone addiction. Further, attachment anxiety, an aspect of insecure adult attachment, was shown to have a mediating effect on the relationship between smartphone addiction and self-directed learning ability. Finally, to strengthen self-directed learning in university students today, different strategies focused on smartphone addiction awareness and on ways to prevent students’ from developing insecure adult attachment should be developed and systematically applied, and campaigns should be created in this regard.

## Figures and Tables

**Table 1:** Participant’s general characteristics (*n* = 235).

Characteristics	Category	*n* (%)
Gender	Male	75 (31.9)
	Female	160 (68.1)
Major	Health studies	98 (41.7)
	Humanities and social science	61 (26.0)
	Natural science	12 (5.1)
	Engineering	40 (17.0)
	Arts and sports	24 (10.2)
Year	1st	52 (22.1)
	2nd	55 (23.4)
	3rd	112 (47.7)
	4th	16 (6.8)
Average grade	A or higher	38 (16.2)
	B	157 (66.8)
	C or lower	40 (17.0)
Average daily smartphone use	4 h or less	83 (35.3)
	4–7 h	139 (59.1)
	8 h or more	13 (5.5)
Daily average study time	2 h or less	49 (20.9)
	2–4 h	100 (42.6)
	4 h or more	86(36.5)
Reason for choosing the major	Aptitude and interest	127 (54.0)
	Better chance of getting a job	58 (24.7)
	Encouraged by parents and others	17 (7.2)
	Based on grade	17 (7.2)
	Valuable job	16 (6.8)
Main smartphone function	Social network, text messaging	177 (75.3)
	Music, movies, games	29 (12.3)
	Information use on Internet	29 (12.3)
How important is your smartphone?	Important	180 (76.6)
	Neutral	55 (23.4)
	Not important	0 (0.0)
Are you addicted to smartphones?	Yes	102 (43.4)
	No	133 (56.6)

**Table 2:** Correlations between smartphone addiction, adult attachment, and self-directed learning (*n* = 235).

Variables	Mean (*SD* ^a^)	Smartphone Addiction *r (p*-Value)	Adult Attachment *r (p*-Value)	Attachment Anxiety *R (p*-Value)	Attachment Avoidance *r (p*-Value)
Smartphone Addiction	2.52 (0.63)	--			
Adult Attachment	2.78 (0.28)	0.47 (<0.001)	--		
Attachment Anxiety	2.64 (0.47)	0.46 (<0.001)	0.84 (<0.001)	--	
Attachment Avoidance	2.94 (0.30)	0.09 (0.189)	0.42 (<0.001)	−0.12 (0.064)	--
Self-Directed Learning	3.37 (0.31)	−0.34 (<0.001)	−0.33 (<0.001)	−0.39 (<0.001)	0.06 (0.40)

^a^*SD:* standard deviation.

**Table 3:** The mediating effect of adult attachment on the relationship between smartphone addiction and self-directed learning (*n* = 235).

Stage	Adult Attachment					Self-Directed Learning					*R* ^2^	Adj *R*^2^	*F*	*p*
	*B* ^b^	*SE*	β	*t*	*p*	*B*	*SE*	β	*t*	*p*				
1. Smartphone addiction → Adult attachment	0.19	0.02	0.46	8.00	<0.001						0.22	0.21	63.92	<0.001
2. Smartphone addiction → Self-directed learning						−0.17	0.03	−0.34	−5.56	<0.001	0.12	0.11	30.88	<0.001
3. Smartphone addiction, Adult attachment →Self-directed learning						−0.12 −0.25	0.03 0.08	−0.24 −0.22	−3.49 −3.23	0.001 0.001	0.16	0.15	21.28	<0.001

*B*^b^*,* unstandardized beta coefficient; *SE,* standard error, Adj, Adjusted.
